# Hallmarks for Thrombotic and Hemorrhagic Risks in Chronic Kidney Disease Patients

**DOI:** 10.3390/ijms25168705

**Published:** 2024-08-09

**Authors:** Zeeba Saeed, Vittorio Sirolli, Mario Bonomini, Sabina Gallina, Giulia Renda

**Affiliations:** 1 Center for Advanced Studies and Technology, G. d’Annunzio University of Chieti-Pescara, 66100 Chieti, Italy; zeebasaeed20@gmail.com; 2 Nephrology and Dialysis Unit, Department of Medicine, G. d’Annunzio University of Chieti-Pescara, SS. Annunziata Hospital, Via dei Vestini, 66100 Chieti, Italy; vittorio.sirolli@unich.it (V.S.); mario.bonomini@unich.it (M.B.); 3 Department of Neuroscience, Imaging and Clinical Sciences, G. d’Annunzio University of Chieti-Pescara, 66100 Chieti, Italy; sabina.gallina@unich.it

**Keywords:** chronic kidney disease, hemostatic dysfunction, hemodialysis membranes, antiplatelets, anticoagulants

## Abstract

Chronic kidney disease (CKD) is a global health issue causing a significant health burden. CKD patients develop thrombotic and hemorrhagic complications, and cardiovascular diseases are associated with increased hospitalization and mortality in this population. The hemostatic alterations are multifactorial in these patients; therefore, the results of different studies are varying and controversial. Endothelial and platelet dysfunction, coagulation abnormalities, comorbidities, and hemoincompatibility of the dialysis membranes are major contributors of hypo- and hypercoagulability in CKD patients. Due to the tendency of CKD patients to exhibit a prothrombotic state and bleeding risk, they require personalized clinical assessment to understand the impact of antithrombotic therapy. The evidence of efficacy and safety of antiplatelet and anticoagulant treatments is limited for end-stage renal disease patients due to their exclusion from major randomized clinical trials. Moreover, designing hemocompatible dialyzer membranes could be a suitable approach to reduce platelet activation, coagulopathy, and thrombus formation. This review discusses the molecular mechanisms underlying thrombotic and hemorrhagic risk in patients with CKD, leading to cardiovascular complications in these patients, as well as the evidence and guidance for promising approaches to optimal therapeutic management.

## 1. Introduction

Chronic kidney disease (CKD) is a major public health issue that affects between 8% and 16% of the population globally [1]. CKD is classified and staged according to the combined association of estimated glomerular filtration rate (eGFR) category (G1–G5) and albuminuria category (A1–A3), with worsening categories being incrementally associated with increased risk of complications [2,3]. By 2030, it is expected that the use of renal replacement therapy will increase to 5.4 million people worldwide, creating major public health issues [4]. 

Cardiovascular diseases (CVDs) are the leading cause of death in CKD patients, with a multifactorial pathophysiology associated with traditional and non-traditional risk factors [5]. In CKD stage 4 and 5 patients, 40–50% of all deaths are due to cardiovascular (CV)-related mortality [5]. The presence of CVD results in the rate of hospitalization being 60% higher among CKD patients compared to those without CVD [6]. End-stage renal disease (ESRD) patients, including those receiving hemodialysis (HD) are predisposed to hemostatic alterations entailing bleeding and thrombotic diathesis which make risk management challenging [7,8]. Thrombotic events in CKD are associated with hemostatic, inflammatory and endothelial dysfunction pathways, involving the activation of procoagulant factors and platelets and the reduction in endogenous anticoagulants and fibrinolytic activity. Moreover, comorbidities including diabetes and dyslipidemia are linked with thrombosis risk in CKD patients [9,10]. In addition, dialysis treatment increases thrombogenicity in CKD patients due to hemoincompatibile dialyzer membranes, which activate the coagulation cascade. On the other hand, bleeding complications in ESRD patients are due to platelet dysfunction, platelet–vessel wall interaction, anemia, accumulation of drugs due to reduced clearance, and anticoagulation during HD [11]. 

Antithrombotic agents, including anticoagulant and antiplatelet drugs, are used in primary and secondary prevention of thrombotic events, but they increase the risk of bleeding complications in ESRD patients [12,13]. Maintaining the balance between the benefits of antithrombotic drugs and bleeding risk has been always an important issue for clinical physicians [14]. Oral anticoagulants are used mainly for the prevention of thromboembolism in patients with atrial fibrillation (AF) and for the prevention and treatment of deep venous thrombosis and pulmonary embolism. Vitamin K antagonist (VKA) oral anticoagulants, inhibiting the hepatic synthesis of thrombin and factor VII, IX, and X, are currently replaced in most conditions by direct oral anticoagulants (DOACs), including the thrombin inhibitor dabigatran and the factor Xa inhibitors rivaroxaban, apixaban, and edoxaban, which have predictable pharmacokinetics and pharmacodynamics, fixed dosing, limited interactions with food and drugs, and no requirement for routine monitoring, as well as a better efficacy/safety profile compared with VKAs [12]. 

Aspirin is the cornerstone antiplatelet therapy in patients with known CVD, providing a net benefit in the secondary prevention of major CV events; in primary prevention, its net balance between benefit and harm is less clear, and it is indicated in patients with high CV risk without an increased bleeding risk. 

P2Y_12_ inhibitors, including clopidogrel, ticagrelor, and prasugrel, further reduce the risk of CV events when added to aspirin (double antiplatelet therapy, DAPT), particularly in high-risk conditions such as acute coronary syndromes (ACS) and percutaneous coronary intervention (PCI) as well as in chronic coronary syndromes, when patients’ characteristics and/or coronary procedural features require a more intense antiplatelet treatment. Because clopidogrel exhibits variability in response, due to genetic mutation and drug interactions, ticagrelor and prasugrel are currently preferred in many conditions. 

Besides the protective effect of antithrombotic therapy for the prevention of thrombotic/ischemic events, the interaction of these drugs with the hemostatic system entails an increase of bleeding risk, which is higher when two antiplatelets are associated, for example, when DAPT is prescribed for ACS, or when one or two antiplatelets are associated with an anticoagulant due to the coexistence of AF and ACS.

Due to the simultaneous existence of prothrombotic and high bleeding risk in CKD patients, personalized and careful clinical monitoring is required before prescription of antithrombotic agents [15]. Moreover, modification of hemodialyzer membranes would increase their performance efficiency, hemocompatibility, and antioxidant and antithrombogenic characteristics, with a subsequent reduction in thrombus formation [16,17,18]. 

This review paper provides a comprehensive overview of the underlying mechanism in CKD-induced hemostatic alterations and the strategies to mitigate thrombotic and hemorrhagic risk in this population, in order to obtain a clear understanding of the risk profile of patients, with subsequent implementation of effective therapies. 

We used different databases, including Science Direct, Google Scholar, and PubMed. Various terms were used for searching articles, which mainly include ‘cardiovascular disease’, ‘platelet activation’, ‘hypercoagulation’, ‘microparticles’ or ‘extracellular vesicles’, ‘thrombosis’, ‘hemorrhage’, ‘antiplatelet therapy’, ‘anticoagulation’, ‘hemodialysis’, and ‘dialysis membrane’ in combination with the term ‘chronic kidney disease’. In addition, connected papers and references of retrieved articles were used to search for additional relevant publications.

## 2. Cardiovascular Diseases in CKD

**Figure 1:**
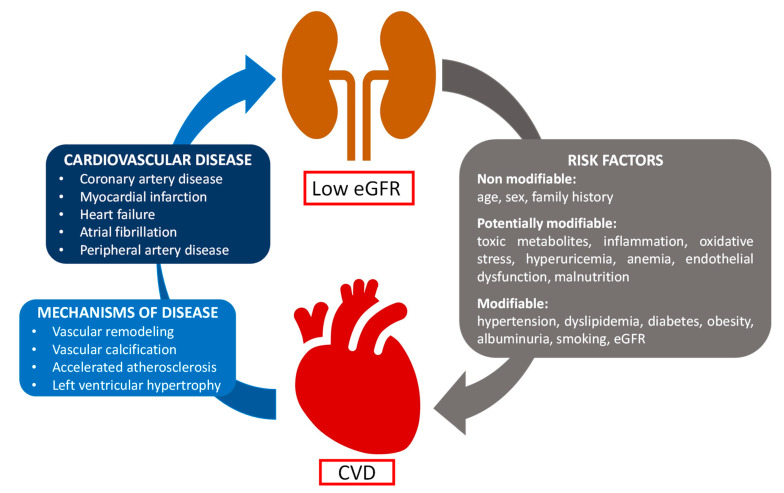
Pathophysiology of cardiovascular diseases in patients with chronic kidney failure. Several risk factors, both modifiable and non-modifiable, contribute to the pathological mechanisms leading to CV events in patients with chronic kidney failure. On the other hand, CVD often contributes to worsened kidney function, accounting for the complex relationship between heart and kidney pathophysiology. CVD = cardiovascular disease; eGFR = estimated glomerular filtration rate.

Hemostatic dysfunction in uremic patients increases CV risk [19]. In CKD patients, the pathophysiology of CVD is multifactorial, involving the formation of thrombus on top of the ruptured atherosclerotic plaque, vessel remodeling, and endothelial dysfunction, and resulting in myocardial infarction, stroke, and thromboembolic events such as deep vein thrombosis and pulmonary embolism [20,21]. The multiple risk factors of CVD in CKD are categorized into non-modifiable risk factors (age, sex, and family history), potentially modifiable risk factors (toxic metabolites, inflammation, oxidative stress, hyperuricemia, endothelial dysfunction, anemia, and malnutrition), and modifiable risk factors (hypertension, dyslipidemia, diabetes, albuminuria, obesity, smoking, and eGFR) [5,22] (Figure 1). The risk of CVD exponentially increases with the progression of CKD [23]. CKD is an independent risk factor for coronary artery disease (CAD), which is the leading cause of morbidity and mortality in patients with CKD. Moreover, coronary angiography carries a risk of contrast nephropathy in patients with advanced CKD, and PCI is associated with higher procedural complications, restenosis, and future cardiac events as compared with the non-CKD population. On the other hand, coronary artery bypass grafting reduces repeat revascularizations but is associated with significant perioperative morbidity and mortality [24].

Heart failure (HF) is a cause of morbidity and mortality in CKD patients; 40% of CKD patients on dialysis develop HF, both with preserved ejection fraction (HFpEF) and reduced ejection fraction (HFrEF) [6]. Yu et al. [6] evaluated the occurrence of HFpEF and HFrEF in CKD patients with eGFR ≤ 45 and compared the attribution of both types of HF in all-cause death and CV death in this population. This study showed a higher risk of all-cause and CV death in CKD with HF compared with those without HF. Further, their finding demonstrated higher prevalence of HFpEF (over 70%) among CKD patients compared with HFrEF, although all-cause mortality and CV death were higher among CKD patients with HFrEF than HFpEF. The study of Cobo Marcos et al. [25] showed that older age, female sex, high prevalence of HFpEF, and comorbidities such as kidney related anemia increase the coexistence of HF and CKD. 

In patients with CKD, AF is a common arrhythmia, accounting for 15–20% of those with severe CKD. Moreover, 40–50% of patients with AF also exhibit CKD [26]. Renal dysfunction, associated with proinflammatory state, endothelial dysfunction, systemic arterial hypertension, and left ventricular hypertrophy, increases the chances of AF. In addition, lower eGFR and increased urine albumin-to-creatinine ratio were significantly linked with higher risk of AF [27]. The prevalence of AF in HD patients increased with age, being 33.2% in patients older than 74 years compared with 24.6% in patients with 65–74 years [28]. Patients with both AF and CKD have a higher risk of stroke, CV morbidity, and all-cause mortality in comparison to those with either CKD or AF alone [29]. In ESRD patients on maintenance HD, the arteriovenous fistula is the preferred vascular access; this has a significant impact on the hemodynamics of the cardiovascular system, with a resultant increase in CVD complications [30]. The study of Song et al. [30] indicated a significant increase in the rate of occurrence of AF in ESRD patients on HD using the arteriovenous fistula compared with those using tunneled-cuffed catheters. After the establishment of the arteriovenous fistula, left atrium enlargement, increased left ventricular mass index, and decreased left ventricular ejection fraction were revealed by echocardiography, followed by a further increase in the occurrence of AF. Further, the author added that age and left atrium enlargement were the independent risk factors for new-onset AF. New-onset AF and left ventricular systolic dysfunction were independent risk factors for adverse clinical outcomes in maintenance HD patients after arteriovenous fistula establishment. The adverse clinical complications, including stroke, HF, CV death, and all-cause mortality, were significantly higher in the AF group compared with non-AF group, revealing a potentially poor prognosis for arteriovenous fistula hemodialysis patients with concomitant AF. An increase in thromboembolic events in patients with AF is associated with left atrium enlargement, which is an independent prognostic value for the occurrence of stroke. 

Peripheral artery disease (PAD) is common in CKD patients and is related to high CV morbidity and mortality [31]. PAD is highly prevalent in CKD patients with stage 3–5 and CKD stage 5 on dialysis compared with patients having eGFR ≥ 60 mL/min/1.73 m^2^, with consequent incident of lower-limb complications [32]. The occurrence of PAD is associated with male sex, older age, diabetes mellitus, inflammation, ischemic heart disease, prealbumin levels, vascular calcification, and C-reactive protein [33]. The study of Hopley et al. [34] showed increased risk of CV death, myocardial infarction, and ischemic stroke in patients with PAD and CKD compared with patients without CKD. To date, the success of building improved models for predicting CVD risk involving CKD has been limited. The inclusion of parameters such as race, renal function, and increased urine albumin excretion did not improve the performance of the Framingham Heart Score model. Predicting circulating biomarkers is important to develop risk factor-driven models, particularly in CKD patients, which involve multifactorial contributors to clinical outcomes [35].

## 3. Mechanisms of Hemostatic Dysfunction in CKD

Both thrombotic and bleeding risk can independently be increased by impaired renal function and elevated albuminuria [35]. Previous studies reported that CKD patients with preserved and moderate renal function had thrombosis risk, whereas ESRD was associated with hemorrhagic events [36,37]. The tendencies of patients to develop thrombotic or bleeding complications are still unclear. However, there are factors increasing both thrombosis and bleeding risks through different mechanisms [38]. Platelet dysfunction in ESRD patients is triggered by the accumulation of uremic toxin, which consequently is responsible for the bleeding risk in these patients. Additionally, anemia, thrombocytopenia, uremic disturbance of coagulation, and fibrinolysis contribute to the bleeding risks in the ESRD population [7]. Ocak et al. [39] reported that CKD patients have a 1.5-fold higher bleeding risk than non-CKD patients, but this risk is not uniform across CKD patients: a 3.5-fold increase in bleeding risk was observed in patients with an eGFR of <45 mL/min/1.73 m^2^ having albuminuria, whereas CKD patients with an eGFR of <45 mL/min/1.73 m^2^ without albuminuria did not exhibit an elevated bleeding risk. On the other hand, some studies reported that in ESRD and CKD stage 1–3 patients in the presence of albuminuria, venous thrombosis is highly prevalent [36,40]. Nopp et al. [41] reported that the increased level of growth differentiation factor-15 (GDF-15) is associated with major bleeding, major adverse CV events, and all-cause mortality, except arterial thromboembolism in ESRD patients on HD. The findings of this study highlighted that GDF-15 is a promising biomarker for bleeding risk prediction in ESRD patients. In addition, GDF-15 enables clinical decision making for dialysis treatment modalities and anticoagulation treatment. Moreover, since patients with a high level of GDF-15 had the highest bleeding risk and lower thrombotic risk, it would be reasonable to not prescribe anticoagulants to this group of patients. 

The factors involved in developing thrombotic risk include blood abnormalities, comorbidities, endothelial dysfunction, and inflammation [42]. Decreased clearance and accumulation of pro-inflammatory substances such as glycation end-products, low defense mechanisms against oxidative stress, and dialysis-associated issues, such as vascular access infections or dialysate back-leak, cause an inflammatory state in CKD. These factors trigger endothelial and platelet activation, with a subsequent increase in the production of coagulation factors by the liver, which generates a prothrombotic condition [43]. 

The risk of thromboembolism in CKD stages 2–3a is 2.5-fold, whereas it increases to 5.5 times higher in CKD stages 3b–4 [44]. Ischemic stroke, myocardial infarction, peripheral artery occlusion, deep vein thrombosis, pulmonary embolism, and vascular access thrombosis are thrombotic complications in CKD patients [45].

The occurrence of thromboembolic complications in CKD patients on dialysis is due to the effect of several risk factors, including the dialysis membrane, arrhythmias, uremic factors, and inflammation of the coagulation system [22,43]. Complex hemostatic alterations with a hyper- or hypocoagulable tendency can occur in ESRD. In the hypercoagulable condition, increased fibrinogen levels and a hypofibrinolytic state are observed, whereas a hypocoagulable condition exhibits reduced thrombin generating capacity and platelet dysfunction [7]. According to previous studies, ESRD patients undergoing HD also experience bleeding complications, ranging between 2.1 and 16.1% per year. Platelet dysfunction, abnormalities in platelet–vessel wall interaction, and administration of the anticoagulants during HD may be the reasons for the risk of bleeding [8]. The different clinical hemostatic symptoms in ESRD patients are due to variation in individual patient characteristics and treatment [45].

The standard coagulation screening has some limitations because it does not assess the whole components of hemostasis [43]. In conventional hemostatic tests, hereditary and acquired coagulation factors and platelet disorders are mostly excluded, which results in limited or no information on global hemostasis [46]. The coagulation system and its complicated relationship within Virchow’s triad is worth exploring because of its key contribution in thrombotic complications. Global coagulation assays, including thromboelastography, calibrated automated thrombogram, overall hemostatic potential, and tissue factor pathway inhibitor, outweigh the conventional coagulation assays because they involve viscoelastic testing for evaluation of coagulation end products (thrombin and fibrin), which can provide a complete overview of the coagulation process, with particular assessment of clot formation and lysis [35].

### 3.1. Endothelial Dysfunction 

The endothelium is involved in maintaining hemostasis via synthesizing and secreting factors such as nitric oxide (NO), von Willebrand factor (vWF), prostacyclin (PGI_2_), thrombomodulin, and tissue plasminogen activator, which control the coagulation cascade, modulate vascular tone, and secrete endothelial microparticles (MPs) [36,38,47]. Endothelial dysfunction and arterial stiffness are the major contributors to CVD in CKD patients [48]. Even patients with mild CKD are prone to the endothelial dysfunction, which promotes vascular stiffness due to alteration in the proinflammatory and prothrombotic microenvironment [36]. 

Under normal physiological conditions, endothelial cells exhibit an anti-adherent and anticoagulant surface; in contrast, damaged endothelial cells expose molecules on their surface, which increase their cell adhesion capacity. The damaged surface triggers platelet binding, with consequent initiation of the coagulation process and development of thrombosis and inflammation, resulting in CV complications [49,50]. The mechanisms of venous and arterial thrombus formation are different. The arterial thrombus formation occurs because of the damaged endothelial cells and exposure of underlying subendothelial matrix and vascular smooth muscle cells [51]. However, the release of endothelial MPs causes venous thrombosis on an intact endothelial layer. Thus, anti-platelet agents and anticoagulant drugs are recommended for arterial thrombosis and venous thrombosis, respectively [52].

Vascular dysfunction and endothelial dysfunction are major contributors in CKD pathophysiology [49]. Endothelial dysfunction induces the production of proinflammatory molecules interleukin-6 (IL-6) and tumor necrosis factor-α, which cause vasculature impairment indirectly by enhancing C-reactive protein synthesis. The increased level of C-reactive protein is associated with increasing risk of ischemic stroke [36]. The increased soluble tissue factor (TF) and high TF activity in CKD and ACS are related with endothelial activation and increased risk of CV death [49]. Endothelial dysfunction causes abnormal release of thrombotic and antithrombotic mediators, including TF, FVIII, thrombomodulin, vWF, endothelial protein C receptor, and protease-activated receptors, which cause hemodynamic alterations correlated with the progression of renal dysfunction, leading to thrombotic and bleeding events [36]. FVIII and vWF are the markers of endothelial damage in CKD; the elevated levels of both these factors consequently lead to venous thrombosis [53]. Endothelial dysfunction stimulates the release of thrombomodulin, which is associated with the progression of CKD stage, with parameters of kidney function including urea, creatinine, and cystatin C, and with hypertension, oxidative stress, and left ventricular hypertrophy [36]. The elevated thrombomodulin level is involved in clot dissolution, with subsequent bleeding risks [54].

NO has vasorelaxant, anti-inflammatory, and antithrombotic properties. NO reduction is a marker of the development of endothelial dysfunction in patients with CKD, which is caused by multiple factors, such as increased levels of endogenous inhibitors of endothelial NO synthase, advanced glycosylation products, phosphate, pro-inflammatory cytokines, oxidative stress, and fibroblast growth factor 23. Decreased protective factors such as Klotho and vitamin D are two additional factors [55]. α-Klotho protein plays a vital role in maintaining the integrity and homeostasis of the endothelial cells through suppressing the expression of the adhesion molecules, such as intercellular adhesion molecules and vascular cell adhesion molecules, inhibiting the signaling pathway of nuclear factor-κB, avoiding hyper-permeabilization, and preventing apoptosis of endothelial cells through the internalization of the complex consisting of canonical transient potential receptor 1 and vascular endothelial growth factor receptor 2. The deficiency of α-Klotho protein in CKD patients predisposes them to greater risk of CVD [50]. 

### 3.2. Platelet Dysfunction

**Figure 2:**
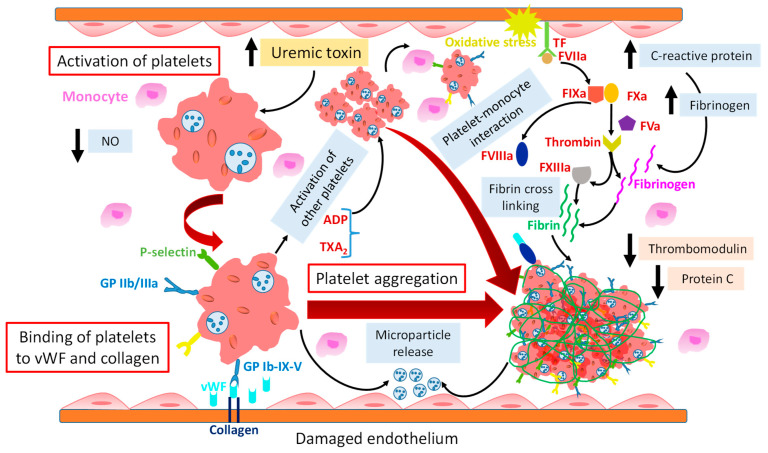
Mechanisms of platelet hyperreactivity in patients with chronic kidney disease. Uremic toxin triggers platelet hyperreactivity, induces release of platelet-derived MPs, results in overexpression of GP IIb/IIIa and P-selectin, and enhances platelet monocyte aggregation. GPIb-V-IX receptors on platelets are involved in platelet adhesion to vWF and collagen, as unveiled on damaged endothelium. Activated platelets recruit other circulating platelets by releasing mediators such as ADP and TXA_2_, leading to thrombus formation. The binding of fibrinogen with platelets is mediated by activated GP IIb/IIIa, which induces platelet aggregation. C-reactive protein is associated with increased fibrinogen plasma level. Exposed TF on the disrupted vessel wall triggers thrombin generation, which consequently converts fibrinogen to fibrin to form a stable clot. vWF exhibits a prothrombotic effect by carrying FVIII and facilitating platelet aggregation and adhesion. Reduced NO level induces platelet activation and aggregation. The decreased levels of thrombomodulin and protein C increase the coagulation tendency and thrombotic risk. ADP = adenosine diphosphate; NO = nitric oxide; TF = tissue factor; TXA_2_ = thromboxane A_2_; vWF = von Willebrand factor; upward black arrow = increase; downward black arrow = decrease.

Platelets play a key role in hemostasis [43], and their hyper or hypo reactivity may be involved in the development of thrombotic or hemorrhagic complications in patients with CKD [56]. High platelet activation and aggregation are key factors in thrombus generation in CKD (Figure 2) [31,52]. vWF and collagen are unveiled on the subendothelial surface upon endothelial dysfunction. Receptors on the platelet surface, such as GPIb-V-IX and GPVI, are involved in platelet adhesion to vWF and collagen, respectively, to promote thrombus formation [56]. The binding of fibrinogen with platelets is mediated by activated integrin α_IIb_β_3_ (GP IIb/IIIa), which induces platelet aggregation. In order to increase thrombus formation, activated platelets recruit other circulating platelets by releasing mediators such as adenosine diphosphate (ADP) and thromboxane A_2_ (TXA_2_). In addition, exposed TF on disrupted vessel walls triggers thrombin generation, which consequently converts fibrinogen to fibrin to form a stable clot [9,56]. However, in CKD patients, the reduced platelet adhesion is followed by endothelial dysfunction, which elevates the coagulation factors and decreases fibrinolytic activity, with a consequent increase in thrombotic risk [38,57]. Clinical studies revealed that platelet activation increases with the progression of kidney impairment [58]. 

Activated platelets recruiting neutrophil enhance the hypercoagulability [59]. CKD-induced atherosclerotic plaque rupture enhances coagulation by recruiting more platelets to vulnerable areas, leading to abnormal platelet aggregation. The aggregated platelets form a highly stable thrombus by binding with other blood cells [36]. 

In CKD patients, the decreased endothelial integrity promotes the reduction of antiplatelet mediators such as glycocalyx, NO, PGI_2_, and CD39/CD73, inducing platelet activation and aggregation. In patients with acute kidney injury, the disruption of glycocalyx triggers endothelial activation, which consequently enhances platelet activation and their adhesion on the endothelium surface. Endothelial dysfunction decreases NO synthesis, leading to an abnormal platelet aggregation. In contrast, uremia patients have elevated plasma NO metabolites, which might decrease platelet aggregation. The action of cyclooxygenase (COX) and prostacyclin synthase on arachidonic acid (AA) releases PGI_2_, which causes the inhibition of platelet aggregation by enhancing the cyclic adenosine monophosphate level in platelets. The dysfunction of PGI_2_ can develop CVD and thrombotic complications. In CKD patients, the reduced level of CD39 increases ADP, which stimulates platelet aggregation. Moreover, the maintenance of an inflammatory condition could prevent abnormal platelet activation in CKD [9]. Cofer et al. [31] reported a higher platelet aggregation induced by ADP, AA, epinephrine, and serotonin in CKD vs. non-CKD patients. Similarly, it was reported by Nishi et al. [60] that higher ADP and collagen-induced platelet aggregation occurred in patients with mild to severe CKD than non-CKD patients. On the other hand, the study of Khalid et al. [46] examined platelet dysfunction in CKD stage 4 and 5 patients in a bleeding context, using light transmission aggregometry, platelet secretion assay, and platelet nucleotide analysis. The heterogeneous nature of patients in terms of dialysis vs. no dialysis, multifactorial bleeding risk, complex hemostatic alteration, and different laboratory procedures for the platelet function test is the cause of unclear patterns of results among these groups.

Gomchok et al. [9] reported that increased TF expression and activity in CKD patients reflects the elevated prothrombotic activity of the endothelium as triggered by uremic toxins, including indoxyl sulfate and indole-3 acetic acid. Indoxyl sulfate triggers thrombosis by platelet hyperactivity, increasing the response to collagen and thrombin, inducing the release of platelet-derived MPs and the overexpression of integrin α_IIb_β_3_ and P-selectin, and enhancing platelet monocyte aggregation. This study suggested that inhibiting TF could be a promising therapeutic target to prevent aberrant platelet activation.

**Figure 3:**
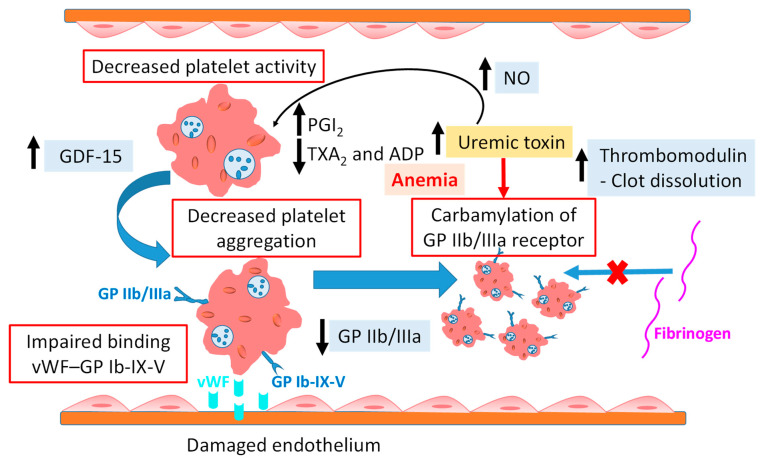
Mechanism of platelet dysfunction in patients with chronic kidney disease. Uremic toxins impair the binding of platelets to endothelial cells via the vWF–GPIb-IX-V receptor complex. The high production of PGI_2_ causes the inhibition of platelet aggregation. In this condition, the numbers and functions of GP IIb/IIIa receptors decline, and reduced production of TXA_2_ and ADP occurs. The carbamylation of GP IIb/IIIa inhibits the binding of fibrinogen with platelets, thereby inducing bleeding. The increased level of GDF-15 is associated with major bleeding. The elevated thrombomodulin level is involved in clot dissolution, with subsequent bleeding risk. The increased plasma NO level in CKD patients decreases platelet aggregation. The synergistic effect of anemia and CKD imposes a worsening effect with regard to platelets abnormalities. ADP = adenosine diphosphate; GDF-15 = growth differentiation factor 15; NO = nitric oxide; PGI_2_ = prostacyclin; TXA_2_ = thromboxane A_2_; vWF = von Willebrand factor; upward black arrow = increase; downward black arrow = decrease.

However, platelet dysfunction in CKD patients also contributed to bleeding risk [43]. Renal impairment causes hemostatic dysfunction, which can be responsible for minor or major bleeding events, from bruising to gastrointestinal, intracranial, and life-threatening bleeding. The pathophysiology of hemorrhage is multifactorial, because bleeding occurs even in the presence of normal concentrations of coagulation factors and normal platelet counts, revealing the role of acquired defective primary homeostasis in hemorrhagic risk [61]. In uremia, the deficient activity of platelets causes extreme bleeding (Figure 3) [52]. Platelet aggregation and adhesion are reduced by uremia through both intrinsic and extrinsic factors. In this condition, the numbers and functions of GP Ib and GP IIb/IIIa receptors decline, and alpha-granule content release from platelets, abnormal metabolism of AA and prostaglandin, COX activity and phospholipase A2 activity, and reduced production of TXA_2_ occur. These factors result in increasing of the bleeding tendency [38,52]. Reduced ADP-mediated platelet aggregation has been observed in CKD patients not on dialysis, particularly in uremic patients [56]. Uremic toxins impair the binding of platelets to endothelial cells via the vWF–GPIb-IX-V receptor complex [54]. Binder et al. [61] examined carbamylation as a mechanistic link between uremia and platelet dysfunction in ESRD patients. Their results revealed inhibition of platelet activation, adhesion, and aggregation by carbamylation through cyanate. Decreased activation of integrin α_IIb_β_3_ was observed in patients on HD as compared to healthy controls. The loss of receptor activity and fibrinogen binding was associated with modification of lysine 185 in the β_3_ subunit, suggesting that carbamylation of GP IIb/IIIa is one of the underlying mechanisms of uremic bleeding. The authors concluded that supplementing free amino acid during dialysis can avoid carbamylation-induced loss of GP IIb/IIIa activity, with consequent maintenance of normal platelet function.

Before dialysis, the prolonged prothrombin time and partial thromboplastin time in ESRD patients increase the bleeding risk, which might be associated with platelet abnormalities [53]. The study of Mitic et al. [8] showed that HD did not significantly enhance the platelet thrombogenicity and neither vWF was linked with it. Further, their study suggested that the lack of availability of the GP receptor in ESRD patients may decrease platelet thrombogenicity in this population.

Platelet volume has been observed to increase with the progression of CKD and increase the risk of CVD complications. Elevated platelet concentration in CKD stage 5 or patients undergoing dialysis can lead to higher CVD risks. It has been reported in the literature that CKD patients without CVD had lower platelet counts than those with CVD [58]. Regardless of platelet count, platelet adhesion and aggregation were reduced on the sub endothelium due to low hematocrit. Thrombocytopenia (platelet count < 150 × 10^9^/L) defects the primary homeostasis and impairs platelet aggregation, with a consequent increase in bleeding risk [62,63].

The thrombotic and bleeding tendency in CKD patients can also be explained by platelet exhaustion, because the high platelet activity induces a secondary loss of platelet function [64]. The discrepancy in data regarding the effect of CKD on platelets could also be due to heterogeneity in disease severity and concomitant comorbidities [56]. Finding the markers of platelet activation and reactivity in plasma or on circulating platelets such as platelet factor 4 and β-thromboglobulin in platelet-poor plasma can help in evaluating the platelet function [65]. The clinical impact of the platelet phenotype in CKD is contradictory and unknown. Understanding the relations among kidney function, platelet activity, and CV complications would enable developing clinical therapeutics by targeting the platelets, with the ultimate goal of reducing CV-associated morbidity and mortality in CKD patients [31,58].

### 3.3. Microparticles

Small-sized vesicles of 0.1–1.0 µm are called microparticles (MPs) [66]. Different cell types, such as platelets, endothelial cells, and leukocytes, upon activation and damage, release MPs, which contain surface proteins and intracellular components from the cell of origin. MPs are considered as biomarkers to estimate thrombosis or inflammatory status [66,67]. In comparison to healthy controls, MPs from uremic patients exhibit higher procoagulant activity [68].

MPs with unique composition and contents differ in their thrombotic potential [69]. Circulating MPs are enriched with TF and phosphatidylserine, which induce thrombogenesis [70]. Elevated TF^+^ MP levels could be the reason for thrombus formation during extracorporeal treatments [68].

The size of MPs helps in evaluating their inflammatory and procoagulant potential, because different-sized MPs exhibit various components and functional activities. Platelet MPs smaller than 0.5 μm trigger the activation of platelets and monocytes, resulting in a prothrombotic condition, particularly by the expression of P-selectin. The diameter of the MPs decreases with increasing plasma creatinine levels, revealing the impaired renal function. Exploring the association of MPs size with different stages of CKD would help in understanding the mechanism of hemostatic complications in CKD and finding promising therapeutic approaches [47].

In CKD patients, multiple factors are involved in the activation of platelets such as accumulated uremic toxins and inflammatory cytokines that trigger the overproduction of microvesicles from platelets [58]. Activated platelets tend to change shape, degranulate, and release MPs in order to bind with other platelets and immune cells [58]. Platelet MPs penetrate the tissue system, which the platelet cells cannot access, causing changes in the physiology of renal cells. Therefore, this provides platelet activation conditions that enhance CKD progression [9]. MPs released by platelets are categorized into three types, including α-granules, δ- granules, and lysosomes. More than 300 types of proteins and bioactive mediators, including P-selectin, thrombospondin, platelet-derived growth factor, thromboxane, and platelet activating factor, are found in platelet MPs. Additionally, messenger RNAs and microRNAs are constituents of platelet MPs, which can be transferred to other cells and modulate gene transcription and protein synthesis, with subsequent involvement in the molecular processes of the oxidative stress, inflammation, and fibrosis of CKD [58]. The level of platelet MPs is higher in HD and peritoneal dialysis (PD) than in healthy people [50]. Uremic toxins make platelets less reactive, reducing the level of granule content [71]. CKD patients with thrombotic complications showed higher levels of platelet MPs [68]. Activated platelets release platelet factor 4 from the α-granules, which has high binding affinity with heparin and heparin-like molecules, reducing antithrombin activity and enhancing procoagulant activity. Subsequently, the binding of antibodies to the heparin–platelet factor 4 complex causes further platelet activation, vessel occlusion, and heparin-induced thrombocytopenia. Investigating levels of platelet degranulation could provide insight on association of platelet stress and dysfunction caused by HD and the uremic toxins with CV complication in ESRD-HD [71].

Activated platelets contain several proteins and cytokines on the platelet surface, including integrin glycoproteins integrin α_IIb_β_3,_ GPIX, and GPIb, as well as platelet activation markers P-selectin and CD40 ligand [9,66].

The level of CD40 ligand from platelet MPs increases with the progression of CKD. CD40 ligand triggers the endothelial cells to release chemokine and expose the adhesion molecules (E-selectin, intercellular adhesion molecule 1, and vascular cell adhesion molecule 1), with the subsequent generation of signals to facilitate recruitment and extravasation of leukocytes, such as macrophages and neutrophils, at the site of injury. This mechanism induces the inflammatory environment in CKD patients [58].

Endothelial MPs are potential biomarkers to identify the endothelial dysfunction and risk of CV complications in ESRD patients [72]. Increased circulating endothelial MPs levels have been associated with low eGFR [72]. Elevated levels of circulating endothelial MPs in CKD patients have been associated with higher mortality [67]. In ESRD patients, the generation of endothelial MPs is induced by uremic toxins (*p*-cresol and indoxyl sulphate), endogenous lipopolysaccharide, advanced-glycation end products, oxidized low-density lipoprotein, cytokines, and other factors [48,68]. Endothelial and platelet MPs may have 50- to 100-fold higher pro-coagulant properties than activated platelets [19].

The findings of Van Bladel et al. [73] revealed a reduced expression of P-selectin in response to ADP, cross-linked collagen-related peptide, and thrombin receptor activating peptide in CKD patients as compared to healthy controls. As P-selectin is found in α-granule, the lower expression of P-selectin could be due to a defect in α-granule release from platelets.

Fonseca et al. [67] investigated the association of circulating MPs such as endothelial-, platelet-, and monocyte-derived MPs with stages of CKD in the presence of CVD, including coronary heart disease, ischemic stroke, and PAD. Their results indicated a high concentration of monocyte MPs with the progression of CKD, whereas no increase in the levels of endothelial and platelet MPs were observed with reduced eGFR. Almquist et al. [74] reported that diabetic patients with CKD stages 3–4 had increased levels of platelet, endothelial, and monocyte MPs compared with diabetic patients without CKD. The results of Lau et al. [69] indicated an increasing trend of endothelial MPs in AF patients with CKD stages, whereas no differences were found in platelet MPs, soluble P-selectin, or E-selectin levels. This study suggested that endothelial MPs are more likely to reflect endothelial dysfunction and its association with renal dysfunction. The study of Amabile et al. [48] aimed to investigate the association between circulating MPs and arterial dysfunction in ESRD patients and examined the cellular origin of the MPs associated with these complications. Their findings indicated a three-fold increase in endothelial MPs, 16.5-fold increase in platelet MPs, and 1.6-fold increase in erythrocytes. However, this study revealed a high correlation of only endothelial MPs with arterial dysfunction; the rest of the MPs did not show any significant correlation. MPs from ESRD patients disrupted endothelium-dependent relaxations and the generation of cyclic guanosine monophosphate. Moreover, endothelial MPs showed about a 60% reduction in the release of endothelial NO, suggesting a strong association of endothelial MPs with endothelial and arterial dysfunction in ESRD. Mörtberg et al. [66] reported increased levels of platelet and endothelial MPs in patients with acute myocardial infarction and CKD compared with those without CKD, despite concurrent DAPT. Their results revealed that platelet MPs exhibiting CD40 ligand were more elevated in CKD 4–5 (median 210/µL) than CKD 3 (142/µL) and normal renal function (101/µL). Similarly, platelet MPs expressing P-selectin were higher in CKD 4–5 (253/µL) than in CKD 3 (147/µL) and normal renal function (106/µL). Furthermore, endothelial MPs expressing E-selectin were higher in CKD 4–5 (245/µL) in comparison with CKD 3 (197/µL) and normal renal function (83/µL).

HD clearance of uremic toxins could theoretically reduce endothelial MP formation [68]. However, the effect of HD on the removal of MPs larger than the pore size of the dialyzer membrane is unclear. On the other hand, some studies reported elevated MPs production during HD due to increased hemodynamic and oxidative stress. Conversely, some studies indicated a low impact of HD on circulating MPs [72,75]. De Laval et al. [68] investigated the effect of HD on the concentration of MPs by evaluating pre-HD and post-HD. Their findings indicated higher plasma levels of platelet MPs (774 vs. 464 × 10^6^ MPs/L), endothelial MPs (764 vs. 683 × 10^6^ MPs/L), and monocyte MPs (337 vs. 216 × 10^6^ MPs/L) during HD compared with pre-HD. The expression of P-selectin and CD40 ligand on platelet MPs and TF^+^ by platelet, endothelial, and monocyte MPs were elevated during HD. Additionally, increased levels of Klotho^+^ and the receptor for advanced glycation end product-positive MPs were observed. In contrast, the study of Ruzicka et al. [75] reported a reduction in levels of platelet MPs (10.4 vs. 18.4 × 10^6^/mL plasma), endothelial MPs (0.59 vs. 0.84 × 10^6^/mL plasma), and leukocyte-derived MPs (1.81 vs. 2.40 × 10^6^/mL plasma) post-HD compared to pre-HD, respectively.

### 3.4. Coagulation Abnormalities

Hemostatic complications due to coagulation abnormalities in ESRD patients is associated with bleeding and thrombotic events [45,76]. The significant alteration of the coagulation system occurs as a result of renal impairment; however, the underlying mechanism involved is not well known [61]. Examining the whole coagulation components and fibrinolytic factors would help in understanding the background of this complex condition [45]. In ESRD patients on HD as well as mild CKD and nephrotic syndrome patients, elevated levels of C-reactive protein, thrombin-antithrombin complex, prothrombin fragment 1.2, D-dimer, fibrinogen, fibrinopeptide A, FVII and FVIII, and vWF indicate intravascular activation of the coagulation cascade [45,77]. The increased levels of these coagulation factors can be due to their increased synthesis and decreased urinary clearance [78]. Conversely, the levels of FIX, FXI, and FXII in ESRD and nephrotic syndrome patients are reduced due to increased urinary secretion [77]. The high level of coagulation factors V, VIII, XIII, vWF, and fibrinogen in CKD significantly determined a hypercoagulability state [54]. vWF exhibits a prothrombotic effect by carrying FVIII and facilitating platelet aggregation and adhesion; indeed, patients with thrombotic complications such as deep vein thrombosis, ischemic stroke, and myocardial infarction have increased levels of circulating active vWF [76]. In the endogenous fibrinolytic system, D-dimer is generated by the action of FXIIIa on monomers and polymers of fibrin through degrading cross-linked fibrin [79]. The increased level of D-dimer is a biomarker for the activation of coagulation and fibrinolytic systems, and it is correlated with microalbuminuria. The elevated level of D-dimer reflects the CV complications and atherothrombosis [80]. The deficiency of anticoagulant proteins, including antithrombin, protein C, and protein S, are involved in causing venous thromboembolism [77].

Increased fibrinogen level is an independent risk factor for atherothrombotic diseases, including ischemic heart disease [45]. Additionally, the study of Chen et al. [81] indicated a significant association of elevated plasma fibrinogen level in PD patients with left ventricular remodeling and CV mortality. Furthermore, the authors concluded that high cholesterol, fasting blood glucose, and high-sensitivity C-reactive protein were associated with increased fibrinogen plasma level in patients undergoing PD. Pénzes et al. [45] explored components such as fibrinogen, FXIII antigen level, FXIII activity, and α_2_-plasmin inhibitor, involved in fibrin formation and stabilization in ESRD patients on hemodiafiltration (HDF) or HD. The results of this study indicated increased levels of fibrinogen and FXIII antigen as well as high FXIII activity and decreased α_2_-plasmin inhibitor activity in ESRD patients. Fibrinogen concentrations in HDF and HD patients were similar, and C-reactive protein was correlated with fibrinogen concentration. The authors concluded that increased concentration of fibrinogen and FXIII in ESRD patients increases thrombosis risk, whereas reduced α_2_-plasmin inhibitor activity contributes to fibrinolytic potential. Decreased fibrinogen clearance due to renal impairment causes increased fibrinogen level in CKD patients [81]. The study of Nosseir et al. [53] showed increased FVIII and reduced protein C activities in ESRD patients on HD, suggesting high coagulation tendency and increased thrombotic risk. Further, the authors explained that the elevated level of FVIII in ESRD patients might be due to renal function loss, which reduces the excretion of procoagulant substances. Van der Vorm et al. [76] examined the level of active vWF in non-dialysis CKD patients, ESRD-HD patients, and ESRD-PD patients. The all-patients groups showed increased levels of active vWF compared with healthy controls (86.4–132.8%). The level of vWF in CKD, ESRD-HD, and ESRD-PD patients was 115.8–179.6%, 122.7–198.4%, and 195.8–275.8%, respectively. The trend of active vWF levels increased with the progression of CKD. With the severity of CKD, the continuous damage and activation of the endothelium enhanced the release of elevated concentrations of ultra-large vWF in circulation. The authors further added that the higher level of vWF in ESRD-PD compared to ESRD-HD patients showed the association of endothelial activation with dialysis modalities. Yu et al. [80] studied the impact of D-dimer level on CKD progression in IgA nephropathy. The findings of this study indicated an increased median plasma D-dimer concentration (220 µg/L FEU) in IgA nephropathy compared with healthy controls (170 µg/L FEU). Reduced eGFR (<60 mL/min/1.73 m^2^), more prevalence of CKD stages 3–4, and elevated levels of proteinuria (>1.0 g/d) were observed in patients with increased concentration of D-dimer. Further, the author concluded that D-dimer is a potential indicator of the thrombotic incidence in CKD patients, whereas its role in the progression of kidney disease is unknown, particularly in patients without thrombotic complications.

Lim et al. [35] investigated the effective biomarkers to predict the thrombotic risk in patients with CKD by evaluating a global coagulation assay in this population in order to correlate the biomarkers to clinical outcomes. Their study involved the collection of blood from patients with eGFR < 30 mL/min/1.73 m^2^ for global coagulation assays that included thromboelastography, calibrated automated thrombogram, overall hemostatic potential, and tissue factor pathway inhibitor. In comparison to healthy subjects, increased amplitude on thromboelastography (70.1 vs. 60.2 mm), higher peak thrombin (233.2 vs. 219.7 mm), and maximum overall hemostatic potential (16.1 vs. 6.4 units) were observed in CKD patients, considering age and gender (mean age 66 years, 36% female). More hypercoagulable parameters were predicted in HD patients than PD patients. Dialysis, elevated fibrinogen, decreased endogenous thrombin potential, higher D-dimer, and increased tissue factor pathway inhibitor were the main predictors of thrombotic complications in thirty-five CKD patients. The authors concluded that decreased thrombin generation and elevated tissue factor pathway inhibitor were linked to high thrombotic risk, suggesting the involvement of complex compensatory mechanisms within the coagulation system, which may be promising to predict clinical outcomes. Mӧrtberg et al. [49] reported that biomarkers such as TF, proteinase activated receptor, soluble urokinase plasminogen activator surface receptor, thrombomodulin, adrenomedullin, renin, and angiotensinogen had an inverse relation with eGFR. The elevated concentration of adrenomedullin indicated the progression of CKD. Urokinase plasminogen activator is an important fibrinolysis activator, which binds to the urokinase plasminogen activator receptors expressed by various cell types. However, soluble urokinase plasminogen activator surface receptors show inflammatory response but exhibit low pro-fibrinolytic properties. Thrombomodulin has vascular protective and anticoagulant characteristics [49]. The renal dysfunction causes reduced clearance of urokinase plasminogen activator/soluble urokinase plasminogen activator surface receptor complexes, which results in fibrinolytic activity by converting plasminogen into plasmin [36]. However, the activation of the fibrinolytic system is inhibited by plasminogen activator inhibitor-1 through inhibiting the tissue plasminogen activator and urokinase. The mechanistic transformation of predominant prothrombotic status to a hemorrhagic condition in CKD patients after thrombolytic treatment is unclear. However, using the exogenous tissue plasminogen activator after ischemic stroke might be one of the reasons for the mechanistic shift from CKD-induced ischemic incidences to post-stroke bleeding complications [36].

### 3.5. Effect of Dialysis Membranes and Modalities

**Figure 4:**
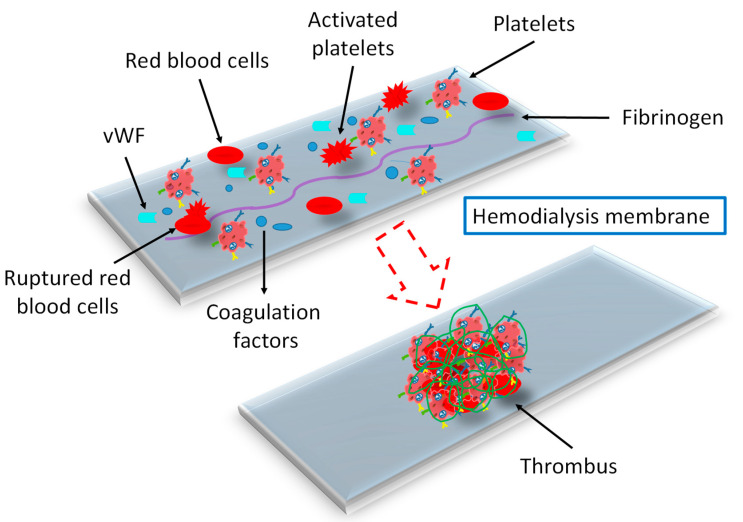
Thrombus formation on hemodialysis membrane after exposure to blood during dialysis. The bioincompatible membrane causes rupturing of red blood cells during HD and induces platelet adhesion and protein adsorption, which induces a coagulation cascade to form a thrombus. After adsorption on the dialyzer membrane, fibrinogen and vWF undergo conformational changes. The conformational changes in fibrinogen and vWF uncover more binding domains to platelets and trigger activation of platelets, blood coagulation, and thrombosis. vWF = von Willebrand factor.

HD and PD are the most common treatments for patients with ESRD to eliminate uremic toxins. Accumulation of these toxic substances causes hemostasis alteration and CV complications [16]. The frequent exposure of blood to the extracorporeal medical device enhances bleeding risk due to absorption of coagulation factors, shear mediated acquired von Willebrand disease, and platelet absorption and dysfunction [82]. Bleeding risk is higher in dialysis patients than in non-dialysis controls; particularly, gastrointestinal bleeding is common among major bleeding events [14]. Various HD procedures may affect platelet function, oxidative stress, and hemostasis in different ways. For these reasons, using different dialysis membranes may also diversely influence the whole hemostasis mechanism, explaining the discrepancy in data related to HD and its influence on platelet function and homeostasis [8]. The hemoincompatible membranes alter the platelet morphology to irregular shape and pseudopods [83]. Rupturing of red blood cells during HD, due to a rough surface, results in the lysis of hemoglobin and platelets and induces platelet adhesion and protein adsorption, which stimulate the coagulation cascade to form a thrombus (Figure 4). The high shear stress during HD triggers the release of platelet MPs. Endothelial MPs concentration was an independent risk factor for CV and all-cause mortality in patients who underwent polysulphone and AN69 dialyzers [68]. Dialyzer membrane stress on platelets increases the release of platelet factor 4 from platelet granules, which enhances thrombus formation by avoiding inactivation of thrombin [17,71]. After adsorption on the dialyzer membrane, the plasma proteins, such as albumin, immunoglobulin G, fibrinogen, fibronectin, factor XII, and vWF, undergo conformational changes, which consequently result in the activation of platelets and leukocytes, activation of the complement system, blood coagulation, chronic inflammation, oxidative stress, and thrombosis [84,85]. The conformational changes in fibrinogen and vWF uncover more binding domains to platelets and leukocytes. The conformational changes in FXII after adhering to the membrane surface trigger the initiation of the intrinsic coagulation pathway to enhance thrombus formation. In certain conditions, conformational changes in fibrinogen may mask the platelet binding site and inhibit platelet adhesion and activation [84,85,86]. Fibrinogen triggers aggregate formation of red blood cells despite low hematocrit levels, which results in defective blood flow and perfusion in the microvasculature during dialysis [85]. The interaction of blood with the membrane, inducing various biological reactions, controls the clotting time, thrombogenic tendency, and the whole mechanism [86]. Nosseir et al. [53] reported that the decreased level of protein C in ESRD-HD patients could be due to the interaction between blood and the dialyzer membrane. Plasma fibrinogen synthesis increases in patients on long-term PD, probably due to the loss of albumin in peritoneal dialysate and the accumulation of fatty acids in blood [81]. The study of De Laval et al. [68] revealed an increased level of platelet, endothelial, and monocyte MPs in patients treated with low-flux compared with high-flux membranes. The authors suggested that the lower production of MPs in high-flux dialyzers could be due to their greater abilities to perform clearance of middle molecules. Complement activation has been reported to be involved in triggering MP release. However, high-flux HD significantly removes complement factors C3a and C5a by filtration and/or adsorption. Uremic toxins also contribute to stimulating MP formation from endothelial cells. The protein binding affinity of these toxins make HD removal less effective, with high discrepancy between different dialyzer types [68].

Van Eck van der Sluijs et al. [87] investigated the impact of dialysis modality on the rate of bleeding risk among 1745 patients: 1211 patients on HD and 534 patients on PD. While taking in consideration age, sex, arterial hypertension, CVD, primary kidney disease, residual glomerular filtration rate, prior bleeding risk, hemoglobin and albumin levels, and antithrombotic and antiplatelet drugs, this study showed 1.5-fold higher bleeding risk for HD compared to PD patients. However, HD patients with previous bleeding history had higher bleeding risk. The author concluded that from a clinical perspective, information about the rate of bleeding risk in HD and PD could enable the physician to choose a preferred dialysis modality for patients according to bleeding risk.

Danielle et al. [88] evaluated the bleeding risk in ESRD patients on dialysis by hemostatic parameters, including platelet count, prothrombin time, and activated partial thromboplastin time (aPTT), according to HD duration. Patients on HD less than 3 months or more than 12 months were included. Bleeding risk was similarly high in HD ≤ 3 month and HD ≥ 12 month groups. Thrombocytopenia was higher in the HD ≥ 12 month group compared with the HD ≤ 3 month group. However, prolonged aPTT was observed in HD ≤ 3 months compared with HD ≥ 12 months group. HD catheter and recent blood transfusion were associated with prolonged aPTT.

### 3.6. Comorbidities

Anemia is related with a developing tendency of thrombosis and bleeding risk as well. In anemic CKD patients, the abnormal activation of PGI_2_, low production of ADP, and altered interaction of platelet–vessel wall increase the hemorrhagic risk. In addition, the increase in NO levels in anemic renal failure hinder platelet aggregation, therefore increasing bleeding tendency in patients [38]. The synergistic effect of anemia and CKD imposes a worsening effect of platelet abnormalities [56]. Reduced erythrocyte number contributes both to bleeding and thrombotic risk by impairing the interaction of platelets with the vessel wall and less scavenging of NO, respectively. Reduction in erythrocyte number with CKD progression resulted from decreased erythropoietin synthesis, inhibition of erythropoiesis by uremic toxin, and lower lifespan of uremic erythrocytes. There is a negative correlation between red blood cell count and bleeding time [56,89].

Using erythropoietin to treat anemia in ESRD patients can reduce bleeding complications, whereas it can also increase the risk of ACS by increasing blood viscosity [38]. On the other hand, Lorentz et al. [82] described that despite being provided erythropoietin and iron supplementations, ESRD patients on chronic HD suffer from anemia due to frequent blood loss from clotting in the hemodialyzers regardless of prophylactic heparinization.

Increased risk of CV events is found in patients with diabetes mellitus and CKD. These two concurrent conditions predispose the patients to thrombotic complications with homeostatic disturbances [10,74]. Patients with diabetes mellitus and CKD have increased platelet-leukocyte aggregation compared with diabetic patients without CKD [74]. In diabetic patients, the alteration in platelet activity is associated with elevated levels of CD39 [90]. In the hyperglycemic condition, the glycosylation of the endothelial system occurs in the kidney, leading to high blood viscosity and causing an osmotic effect on platelets, with subsequent enhancement in platelet reactivity and their interaction with the endothelium [9]. The increased levels of MPs in diabetic kidney disease are associated with multiple factors, such as hyperglycemia, dyslipidemia, and oxidative stress. Previous studies reported increased levels of platelet and endothelial MPs in patients with diabetic nephropathy [10]. P-selectin was higher in diabetic patients with nephropathy than without nephropathy [91]. Yu et al. [10] reported that phosphatidylserine-positive MPs played a key role in procoagulant activities in diabetic kidney disease through interacting with phosphatidylserine positive cells, shortening coagulation time and dramatically increasing FXa/thrombin generation and fibrin formation. In addition, this study showed little effect of TF^+^ MPs in procoagulant activities in diabetic kidney disease.

CKD patients exhibit a particular lipid profile, known as “uremic dyslipidemia”, that can be defined by decreased high-density lipoprotein cholesterol, low-density lipoprotein cholesterol within the normal range, and increased triglyceride levels [92]. Dyslipidemia causes hemostatic alterations. Multiple lipid abnormalities are involved in CV complications in CKD [93]. The study of Barbagelata et al. [94] demonstrated a positive correlation between lipoprotein (a) and CV risk and mortality in CKD patients. Further, the author suggested that lipoprotein (a)-lowering drugs should be explored to reduce CVD risk in all stages of CKD. Lu and Liao [95] reported a significant association of hyperlipidemia with an increased risk of deep vein thrombosis in ESRD patients. In renal failure patients, hyperlipidemia negatively affects platelet function [9]. In dyslipidemia, oxidized phospholipids from low-density lipoprotein particles activate platelets through a specific pattern recognition receptor known as GPIV to promote thrombotic events [96].

## 4. Strategies to Mitigate Thrombosis in CKD

### 4.1. Antiplatelet Therapy

Antiplatelet agents have been used widely to mitigate platelet activation to prevent CV complication in CKD patients. However, the risks and benefits of antiplatelet therapy vary in this population, who are at both thrombotic and bleeding risk. Aspirin performs its antithrombotic action by acetylation of the platelet cyclo-oxygenase (COX)-1, preventing the binding of AA to COX-1 and resulting in the irreversible inhibition of platelet-dependent thromboxane formation. Binding of P2Y_12_ inhibitors to platelet ADP receptors results in the inhibition of ADP-induced platelet aggregation [13]. In CAD, DAPT, including aspirin and P2Y_12_ inhibitors, is used to avoid thrombotic complications [97]. Mann et al. [98] investigated the effectiveness of aspirin in the primary prevention of CVD in CKD patients. The International Polycap Study-3 (TIPS-3) enrolled patients without CV complications, grouped according to eGFR (eGFR < or >60 mL/min per 1.73 m^2^) and treated with aspirin 75 mg daily or a placebo. The results indicated no significance in terms of interaction of eGFR with aspirin versus placebo. However, grouping the whole cohort according to tertiles of eGFR at baseline, larger treatment effects with aspirin were observed at lower eGFR levels: aspirin treatment determined a 38% CVD reduction in the lower eGFR tertile group (eGFR < 70 mL/min per 1.73 m^2^) without increasing bleeding and other severe events, suggesting that low-dose aspirin could be suitable in the primary prevention of CVD in CKD patients with acceptable bleeding risk. On the other hand, Oh et al. [99] reported that low-dose aspirin increases the risks of CV complication in CKD patients, particularly with low body weight (<60 kg), suggesting that the prescription of low-dose aspirin for reducing CV events in CKD patients should be personalized. Similarly, Haim-Pinhas et al. [100] reported that chronic aspirin treatment did not improve survival among elderly patients with CKD without prior CVD. Aspirin therapy is considered a well-established treatment for the secondary prevention of CVD, whereas its role in the primary prevention of CVD in CKD patients is not well validated. The benefit of aspirin therapy in the primary prevention of CVD among CKD needs to be further explored, because multiple risk factors contribute to CVD in CKD patients, including increased oxidative stress, endothelial dysfunction, vascular calcifications, and increased fibroblast growth factor 23. A systematic review and meta-analysis reported that antiplatelet therapy resulted in a 15% reduction of CV events, with no significant impact on all-cause mortality and kidney failure complications. Nevertheless, antiplatelet drugs elevate the risk of major and minor bleeding, although 23 major CV complications could be prevented by antiplatelet therapy among 1000 CKD patients, with the occurrence of 9 major bleedings, suggesting a net clinical benefit of antiplatelet treatment of CKD patients [101]. Jain et al. [102] evaluated platelet aggregation in stage 4–5 CKD patients who were not on dialysis and were asymptomatic for CVD, treated with aspirin 81 mg daily in association with ticagrelor 90 mg twice daily or clopidogrel 75 mg daily for two weeks. Their results indicated a higher inhibition of whole blood platelet aggregation by ticagrelor-DAPT (87 ± 22%) compared to clopidogrel-DAPT (63 ± 50%). Further, this study revealed that ticagrelor-DAPT greatly decreased the plasma levels of anti-inflammatory cytokines such as IL-6 in comparison to clopidogrel-DAPT (8.42 ± 1.73 pg/mL vs. 18.48 ± 26.56 pg/mL, respectively), suggesting that ticagrelor-based DAPT might reduce the inflammation in asymptomatic CKD patients with stage 4 or 5.

Yu et al. [103] reported that CKD patients undergoing PCI had a higher risk of ischemic and bleeding events than those without CKD. P2Y_12_ inhibitor monotherapy after 1–3 months of DAPT was associated with a significantly lower risk of major bleeding compared to DAPT, regardless the presence of CKD, without a benefit in terms of the prevention of major adverse CV events. On the other hand, Kim et al. [104] evaluated the impact of standard DAPT (6–12 months) and prolonged DAPT (12–24 months) on clinical outcomes in patients with CKD who underwent PCI with a drug-eluting stent. Prolonged DAPT showed a reduction in all-cause death and composite ischemic events, with increased risk of bleeding events. However, there was no significant relationship between bleeding risk and renal dysfunction. This study suggested that in order to balance ischemic and bleeding risk in CKD, tailored decisions for DAPT duration should be taken into consideration. Despite multiple studies, no optimal strategy for DAPT has been developed for routine clinical practice in patients with CKD because of variable study designs, populations, and primary results as well as the exclusion of high-risk patients such as those with CKD [105]. Hwang et al. [105] compared the efficacy and safety of short-term (3 or 6 months) vs. prolonged (≥12 months) DAPT after implanting a drug-eluting stent in CKD patients. However, their results indicated no significant difference in clinical outcomes such as all-cause death, myocardial infarction, thrombotic events, stroke, and TIMI (thrombolysis in myocardial infarction) major bleeding between short-term and long-term DAPT. Prolonged DAPT resulted in increased TIMI major bleeding in non-CKD, but it was not significantly different to that of CKD patients. Porlán et al. [106] studied the influence of CKD on the pharmacokinetics and pharmacodynamics of ticagrelor in ACS patients. Based on the estimated renal clearance, patients were categorized into two groups, including patients with eGFR ≥ 60 mL/min and eGFR < 60 mL/min. Platelet function was examined after loading of ticagrelor dose and at discharge by the VerifyNow system at baseline. Moreover, within 1 h of loading dose, the level of ticagrelor and its active metabolite (AR-C124910XX) were analyzed. The results indicated no difference in platelet inhibition between the two groups. However, in comparison to normal renal function, the level of ticagrelor and its active metabolite were higher in CKD patients.

The data on antiplatelet therapy in CKD are of low quality due the discrepancies in trials, subgroup data, and methodological limitations, showing uncertainty of the results. Further studies are needed to understand the mechanisms involved in antiplatelet therapy in CKD patients [99].

### 4.2. Anticoagulant Therapy

CKD and AF patients are predisposed to prothrombotic conditions and increased risk of ischemic stroke. Anticoagulation is widely used for the primary and secondary prevention of thromboembolic events, but the balance between the efficacy and safety of these drugs in CKD patients should be carefully evaluated [22,82,107], because they are significantly influenced by the kidney function [108]. In order to choose the appropriate anticoagulant therapy, the accurate evaluation of renal function is essential. Using the Cockcroft and Gault (CG) formula and the Chronic Kidney Disease Epidemiology collaboration (CKD-EPI) formula may differentiate the subgroup of patients with severe renal impairment and various risks for bleeding. Therefore, the discrepancy of these two established formulas could significantly affect the evaluation of bleeding risk and management of anticoagulant therapy [109]. Catella et al. [109] assessed the renal function of patients with severe renal impairment based on eGFR < 30 mL/min according to the CG formula and eGFR < 30 mL/min/1.73 m^2^ according to the CKD-EPI formula. The patients with severe renal failure were categorized as CG, CKD-EPI, and CG + CKD-EPI. This study aimed to determine the ratio of patients considered to have severe renal impairment according to each formula and risk of major bleeding during the first three months of anticoagulant treatment. After 3 months of anticoagulant therapy, major bleeding, including intracranial bleeding, gastrointestinal bleeding, urinary bleeding, subcutaneous hematoma, muscular hematoma, menorrhagia, and others, were evaluated. A higher rate of major bleeding was observed in the three groups of patients with severe renal failure compared with those with no severe renal impairment. The findings showed a significantly mismatched proportion of patients having venous thromboembolism with these two commonly used formulas and suggested that the combined use of both formulas may identify more venous thromboembolism patients with an increased risk of bleeding.

Warfarin is commonly used in patients with AF and CKD with eGFR < 30 mL/min/1.73 m^2^ due to the lack of evidence of efficacy/safety balance for DOACs in patients with severe renal failure. Warfarin has been previously reported to reduce the risk of ischemic stroke in CKD patients with eGFR < 30 mL/min/1.73 compared with untreated patients; however, it increased the risk of bleeding. The quality of warfarin treatment drastically declines with reducing eGFR. Patients with AF and CKD 3–5 having high individual time in the therapeutic range show a reduced risk of ischemic stroke, major bleeding, and death. Individual time in the therapeutic range ≥ 70% indicates a better warfarin safety profile. In order to reduce the risk of adverse events, CKD patients on warfarin should be closely monitored [110].

There is limited evidence of the benefit–risk balance of DOAC therapy in patients with ESRD from randomized controlled trials; however, observational studies and sub-analyses from randomized controlled trials confirm the use of DOACs in moderate renal failure. The elimination rate of DOACs by the kidney is 27% for apixaban, 36% for rivaroxaban, 50% for edoxaban, and 80% for dabigatran. The accumulation of these drugs due to reduced kidney clearance could cause adverse bleeding effects [107]. For these reasons, dabigatran 150 mg twice daily or 110 mg twice daily is alternatively used according to thrombotic and bleeding risk but cannot be prescribed for eGFR < 30 mL/min/1.73 m^2^, and the anti-Xa inhibitors have to be prescribed according to dose reduction indications. Apixaban 5 mg twice daily has to be reduced to 2.5 mg twice daily if two out of three of the following criteria of weight ≤ 60 kg, age ≥ 80 years, and serum creatinine ≥ 33 mmol/L (1.5 mg/dL) are fulfilled or the single criterion of creatinine clearance 15–29 mL/min is met; rivaroxaban 20 mg once daily has to be reduced to 15 mg once daily if creatinine clearance ≤ 15–49 mL/min; edoxaban 60 mg once daily reduced to 30 mg once daily if weight ≤ 60 kg or creatinine clearance 15–49 mL/min or with concomitant therapy with a strong P-Gp inhibitor [111].

The study of Sin et al. [112] examined the plasma rivaroxaban level in patients with CKD stage 1–3 with AF after receiving 15 or 20 mg daily. Their findings indicated significantly a higher trough level of rivaroxaban in patients who received 15 mg daily. In addition, the mean peak level was higher in the 15 mg group compared with the 20 mg group. Patients receiving 20 mg daily rivaroxaban exhibited an inverse correlation of the plasma trough level with eGFR, and CKD stage 3 patients demonstrated a significantly higher plasma tough level than CKD stage 2 or 1. Patients with bleeding complications exhibited a higher plasma trough rivaroxaban level than those with no bleeding complications. Based on these findings, this study suggested that measurement of plasma trough rivaroxaban levels can help in optimization of dosage in CKD patients. Even at a lower dose of 10 or 15 mg daily, rivaroxaban accumulates in CKD and ESRD patients and is poorly cleared by HD [113].

Wang at al. [114] investigated the impact of ESRD on apixaban pharmacokinetics and pharmacodynamics and examined the ability of HD in the clearance of apixaban from systemic circulation. A 36% higher apixaban exposure in ESRD patients on dialysis than healthy controls manifested the reduced clearance of apixaban in this group. A 4 h HD session resulted in 14% decrease in apixaban exposure in ESRD. The limited clearance of apixaban by HD in ESRD patients suggested that HD is not useful in the removal of apixaban from the systemic circulation in the case of overdose. Apixaban binds to hemoglobin due to its ability to cross red blood cells. There is a significantly inverse relation between hemoglobin concentration and apixaban peak plasma levels. Taking into consideration the prevalence of anemia in CKD patients, this observation should be noted to prevent the bleeding risk associated with apixaban overdosing [115].

The study of Skripka et al. [116] reported that there was a high interindividual variability in trough plasma concentrations of dabigatran. Plasma trough concentration/dose ratio significantly increased in patients with CKD stage 3b, which was affected by older age and comorbidities.

Zagoridis et al. [108] found in a systematic review that apixaban reduced the bleeding risk in ESRD patients with AF and venous thromboembolism compared to warfarin. However, the risk of thrombosis was not significantly different in both arms. On the other hand, Parada et al. [117] reported that CKD patients with eGFR < 30 mL/min/1.73 m^2^ exhibited higher major bleeding risk than ischemic stroke. Anticoagulation negatively influences these patients, causing increased bleeding risk without reducing embolism.

In the Valkyrie study, 132 patients on HD with AF were treated with a low dose of rivaroxaban. This treatment decreased the occurrence of fatal and nonfatal CV events, particularly bleeding risk, in comparison to VKAs [118]. The comparison of apixaban versus warfarin in patients with kidney failure and AF undergoing dialysis indicated that apixaban reduced bleeding rate and reduced the risk of thromboembolism and mortality [119]. In contrast, another study reported no difference in bleeding rate and stroke while using apixaban and warfarin [120].

Fordyce et al. [121] studied the efficacy and safety of rivaroxaban vs. warfarin in patients with renal failure. Their results indicated that rivaroxaban is more suitable than warfarin due to its lower risk of severe bleeding.

The systematic review conducted by Kao et al. [122] reported that VKAs showed higher major bleeding risk than DOACs (dabigatran, rivaroxaban, apixaban 2.5/5 mg twice daily) and no coagulation in ESRD patients on dialysis. There was no difference in the efficacies of VKAs, DOACs, and no anticoagulation for the prevention of thromboembolism. However, dabigatran and rivaroxaban demonstrated fewer embolic events. No differences were shown in all-cause death among VKAs, DOACs, or no anticoagulation.

The limited evidence of the potentials of oral anticoagulation in CKD patients further requires intensive research on the anticoagulants for their effective and safe use in this population [22].

Maintaining renal function in patients co-exhibiting CKD and AF is a crucial objective, because anticoagulation can lead to an impaired renal function, which is known as anticoagulant-related-nephropathy [27,123]. It has been reported by Brodsky et al. [124] that patients with AF and CKD showed a faster decline of renal function when receiving warfarin in comparison to those with no warfarin exposure. Yao et al. [125] found that, compared with warfarin, dabigatran, rivaroxaban, and apixaban caused fewer adverse renal effects, including a 30% decrease in glomerular filtrate compared to baseline acute kidney injury events and a doubling of serum creatinine levels compared to baseline. The use of rivaroxaban in patients with moderate to severe CKD improves and stabilizes eGFR and cardiac valve calcifications as compared to warfarin due to the reduction of cytokine levels [27]. A study conducted by Trevisan et al. [126] showed that the safety of DOACs and VKAs is still questionable, particularly in terms of kidney disease or injury. Their findings indicated a lower risk of kidney impairment and a sustained 30% reduction in kidney function by DOACs compared to warfarin. In addition, DOACs reduced the risk of major bleeding, but the risk of ischemic stroke, systemic embolism, and death were similar in both anticoagulant groups. Further studies are needed to better investigate this issue [117].

In patients with CKD and concomitant AF and CAD, the combination of antiplatelet and anticoagulant drugs could be needed, particularly in ACS or in patients undergoing PCI, at the cost of a high bleeding risk. The randomized clinical trials comparing dual (DAT) vs. triple (TAT) antithrombotic strategy in ACS/PCI patients showed reduced bleeding with DAT and similar incidence of major adverse CV events, although these trials were not powered to assess efficacy outcomes. However, these trials included only a small number of patients with CKD and low eGFR, and evidence regarding the safety of antithrombotic therapy in patients with CKD remains limited. A large-scale retrospective cohort study of Lee et al. [127] considered oral anticoagulant monotherapy as a more suitable antithrombotic therapy than the combined therapy of an oral anticoagulant plus antiplatelets in patients with CKD with concomitant AF and CAD without undergoing PCI. The combined therapy caused increased risks of ischemic stroke, acute myocardial infarction, and hemorrhagic stroke, consistent across all CKD stages. DOACs users exhibited reduced risks for all-cause mortality, acute myocardial infarction, and gastrointestinal bleeding compared to warfarin. However, no difference was observed in stroke risk between the two subgroups. Antiplatelet resistance causes increased thrombogenicity, which could be the possible reason for higher risks of ischemic stroke and acute myocardial infarction despite using combination therapy. Therefore, the author suggested the appropriate use of an oral anticoagulant to overcome the low effectiveness of antiplatelets in patients with CKD with concomitant AF and CAD, particularly in those for whom PCI for coronary revascularization is not favorable.

The management of patients undergoing HD with concomitant AF is complicated and controversial, with a lack of availability of ideal clinical treatment guidelines. In clinical practice, a multiprofessional team is recommended, to provide a personalized treatment for these patients. The optimal AF management strategy mainly includes the prevention of thromboembolic and CVD events and the improvement of symptom treatment with controlling of the ventricular rate and rhythm. Multicenter studies with a high number of patients and multiple parameters will be needed to definitively establish clinical pathways to improve the prognosis of AF patients on maintenance HD with arteriovenous fistula access [30].

Anticoagulation with unfractionated heparin (UFH) and low-molecular-weight heparins (LMWHs) are used to prevent clotting of the HD extracorporeal circuit and optimize HD adequacy. Heparin reduces patient blood loss due to blood entrapment in the HD circuit, as well as the incidence of circuit changeouts resulting from complete dialyzer occlusion. However, approximately 10% of ESRD patients do not receive heparin during HD due to heparin resistance, heparin intolerance, increased bleeding risk, or clinical preferences. In particular, patients undergoing HD have an increased risk for major bleeding events due to heparin exposure, uremic platelet dysfunction, and other comorbidities, especially those treated with UFH. Another potential risk associated with chronic heparin use, UFH and LMWH alike, in HD is heparin-induced thrombocytopenia. Some heparin dosing strategies suggest actively monitoring anticoagulation during HD using laboratory or point-of-care testing; the utility of these monitoring strategies has long been disputed [128].

Huang et al. [14] reported that anticoagulant use in HD patients results in higher gastrointestinal bleeding risk in comparison to the PD group. Therefore, the author suggested that PD is preferable when anticoagulation is required.

### 4.3. Biocompatible Hemodialyzer Membrane

The membrane’s biocompatibility is defined by properties such as morphology, roughness, charge, crystallinity, chemical composition, hydrophilic and hydrophobic regions, adsorption of water, proteins, and ions, influencing solute removal and interactions with blood components [129]. The rough surfaces of the dialyzer membrane trigger hemoincompatibility issues such as induce complement, leukocytes, and coagulation bioactivation [83,86]. Hemoincompatibility and poor permeability of unmodified cellulose membranes have reduced their utilization over the last couple of decades. To address the limitation of cellulose-based membranes, other synthetic membranes, including polymethylmethacrylate, polyacrylonitrile polymer, polysulfone, and polyethersulfone (PES), have been developed. Among these membranes, the demand of polysulfone and PES increased in clinical dialysis because of better hemocompatibility and lower mortality. The contact of blood with synthetic membranes still causes hemostatic complications [84,85]. The adhesion of human serum proteins such as albumin and fibrinogen on the dialyzer membrane should be prevented to avoid thrombosis [85]. Modification of the dialyzer membranes can improve their hemocompatibility and performance efficiency [86] (Table 1). However, to improve the hemocompatibility characteristics of membranes such as antithrombotic, antifouling, and antiadhesion properties, heparin and vitamin E were used [84]. In contrast, some studies reported that supplementation of exogenous vitamin E shows no effect on oxidative stress and rather acts as a pro-oxidant with diverse biological conditions when its accurate administration timing is uncertain. Therefore, designing an anti-oxidative HD membrane could be a promising approach to eliminate reactive oxygen species [130]. The hemolysis ratio indicates the degree of hemocompatibility of the membrane. Based on the hemolysis ratio, membranes are classified into three categories: a hemolytic membrane exhibiting >5% hemolysis, slightly hemolytic 2–5%, and non-hemolytic <2% [16]. The hydrophobic characteristic of the polysulfone membrane leads to blood coagulation cascade, which limits its application [131]. The increased hydrophilicity of the dialyzer membrane reduces protein adsorption and prevents activation of coagulation cascade [83]. Activation of coagulation cascade causing thrombus formation is not only harmful for the patients but also reduces blood flow, decreasing HD efficiency. Thrombus formation on material is triggered by the intrinsic coagulation pathway. Membranes showing prolonged aPTT reveal inhibition of the intrinsic pathway, avoiding thrombus formation on the surface and reducing the need for anticoagulants, with a consequent reduction in hemorrhage risk. However, membranes with positive surface potential inactivate factor V, which could inhibit the extrinsic coagulation pathway [130,132]. The CKD patients undergoing dialysis develop CKD-related anemia, diabetes, and other comorbidities. Therefore, establishing a biocompatible membrane to avoid the negative impact on medication is essential [133]. Wei et al. [83] addressed the complications of endothelial dysfunction during HD treatment. In order to solve this issue, a PES membrane was coated with a glomerular endothelial cell-like coating, which was a hydrated layer prepared from tannic acid (TA) and α-lipoic acid (α-LA). The anticoagulant characteristics of this membrane mimicked glomerular endothelial cells. Their findings indicated the hydrophilic characteristic of the modified membrane, with a contact angle of 29.7°, higher rate of urea removal and bovine serum albumin (BSA) protein retention, and reduction in platelet adhesion by 98.7%, with prolonged plasma recalcification time (PRT). Moreover, the modified membrane exhibited promising hemocompatibility and free radical scavenging ability. The authors concluded that this membrane could be a potential candidate for novel HD membranes to promote dialysis efficiency and reduce coagulation incidence. The work of Xu et al. [130] focused on improving the antioxidant characteristics of the HD membrane. In this study, a PES membrane embedded with hydrogel was grafted with tannic acid (PES/G/E-TA). This membrane showed promising antioxidant behaviors, favorable hemocompatibility with the attenuating intrinsic coagulation pathway, and insignificant impact on blood cells and the complement system. Moreover, the membrane displayed a high clearance of urea and lysozymes as well as albumin rejection, suggesting it could be a suitable antioxidative membrane for clinical HD. In order to efficiently remove protein-bound uremic toxins, Liu et al. [17] modified a polysulfone membrane by incorporating polyethyleneamine, TA, and bimetal ions, which exhibited high affinity for hippuric acid, *P*-cresol sulfate, and indoxyl sulfate. The high adsorption capacity of this membrane is attributed to the π-π or cation-π with aromatic rings of uremic toxins. The results revealed 50% removal of *P*-cresol sulfate and indoxyl sulfate from BSA. The lower concentration of platelet factor 4 by the membrane indicated its prohibition of platelet activation, and prolonged aPTT and prothrombin time demonstrated the increase of both intrinsic and extrinsic coagulation time. Further, the reduced concentration of fibrinogen on the membrane revealed its antithrombogenic properties, suggesting its potential application in HD. Hou et al. [134] reported that in CKD patients, accumulation of exogenous and endogenous toxins increase the burden on the kidney. This study focused on developing plasma separation adsorption membranes called adsorption resin/PES (AR/PES) for the clearance of middle molecular toxins. Their findings indicated 93.4% lysozyme adsorption capacity, high albumin retention, and no hemolysis, showing excellent hemocompatibility. Zhi et al. [132] addressed the challenges of reducing the efficiency of oxygenators during HD due to membrane fouling by the adhesion of blood proteins and blood cells, causing blockage of membrane pores and preventing O_2_ transfer and CO_2_ removal. In this study, the PES membrane was modified by incorporating polyvinylpyrrolidone (PVP) and poly acrylic acid (PAA). Their results indicated lower adhesion of BSA, platelets, and red blood cells on modified PES with no hemolysis as compared to pristine PES, suggesting it could reduce the oxygenator replacement caused by membrane fouling. Abdelrasoul and Shoker [86] compared the hemocompatibility between unmodified PES and PVP-modified PES fabricated by UV-assisted photochemical synthesis. In order to achieve various degrees of PVP modification on each membrane, they were immersed in viscous polymer suspension for 1–4 min and were named PES-PVP1-4 membranes. PES-PVP3 and 4 membranes exhibited lower surface roughness and magnitude, thereby attenuating the release of complement components, including C5a and C5b-9, showing higher biocompatibility than PES-PVP1 and 2 membranes. The active complement system consequently triggered activation of monocytes and release of inflammatory cytokines such as IL-α, IL-β, and IL-6. Moreover, modified PES membranes demonstrated higher antifouling activity against fibrinogen adsorption and interaction compared to unmodified PES due to hydrolyzed and hydrophilic properties, lower surface roughness, and decreased surface charge characteristics. Azhar et al. [18] modified a cellulose acetate membrane (CA) with polyvinyl alcohol (PVA) and polyethylene glycol (PEG). The CA-PEG-VA membrane exhibited favorable performance efficiency and biocompatible characteristics by removing 93% urea and 89% creatinine, lower platelet adhesion and hemolysis ratio, prolonged clotting time, and reduced thrombus formation. Yang et al. [16] addressed the need for highly efficient adsorbents to effectively remove uremic toxin to reduce the economic and societal burden of dialysis treatment. Their findings indicated 1.8 times higher creatinine clearance by the newly prepared nanostructured porous carbon nanofibers with nitrogen-doped zeolites (NZ-PCNF) compared to PCNF. However, the hemolysis rate was >2% for both nanofiber composites. The results of this study suggest that NZ-PCNF can promote efficacy and efficiency of creatinine clearance while maintaining high levels of biocompatibility and hemocompatibility. Voigt et al. [133] compared the removal of middle molecules involved in the pathology of uremia by a PES-based medium cut-off membrane dialyzer (Theranova) in HD with a PES membrane dialyzer (Polyflux 210 H) in HD and HDF modes and a polysulfone membrane dialyzer (FX CorDiax 800) in HDF mode. The findings of this study indicated higher removal of middle molecules by the medium cut-off membrane than high-flux dialyzers. Further, this study investigated the impact of the increased pore size of medium cut-off membranes on the retention of common medications such as erythropoietin, heparin, insulin, and vancomycin and coagulation factors including II, VII, and X, antithrombin III, and protein C in dialysis patients in comparison to the high-flux dialyzers. No increased permeability of the medication and coagulation factors was observed due to high pore size in the medium cut-off membrane, suggesting no requirement for alteration in drug dosing or anticoagulation procedure, while the medium cut-off membrane dialyzer in HD mode compared with polysulfone and PES dialyzers in HD or HDF modes.

Patients undergoing HD are highly affected by inflammation, CV complications, and immunologic dysfunction, which increase their hospitalization, morbidity, and mortality rate. Therefore, modifying HD membranes to enhance their hemocompatibility can improve patients’ outcomes. Evaluating the side effect of dialyzer membranes can help in optimizing dialysis treatment and improving patients’ well-being [85].

## 5. Bioavailability of Drugs

The high susceptibility of CKD patients to adverse drug reactions limits their inclusion in many clinical trials. The increased accumulation of uremic toxin with deteriorating renal function can alter drug bioavailability, efficacy, and safety. Older age and polypharmacy cause adverse drug reactions in CKD patients [135]. Acute kidney injury and bleeding were the most common events of adverse drug reactions. However, the major risk factor for adverse drug reactions was reduced eGFR in CKD. The severe consequences of adverse drug reactions in CKD leads to hospitalization, life-threatening events, or death [135]. The prospective cohort study led by Laville et al. [135] evaluated the relationship between eGFR and adverse drug reactions in a large cohort of CKD patients. Their findings indicated higher incidence of serious adverse reactions, such as drug-induced hemorrhage and acute kidney injury, in patients with eGFR < 30 mL/min/1.73 m^2^ compared with eGFR ≥ 30 mL/min/1.73 m^2^. Antithrombotic agents and renin-angiotensin system inhibitors and diuretics were mainly responsible for the adverse drug reactions. Drug-induced acute kidney injury and hemorrhages accounted for 90% of the adverse drug reactions with interactions. Simultaneous utilization of multiple diuretics, the combination of a diuretic with renin-angiotensin system inhibitors, and the combination of nonsteroidal anti-inflammatory drugs with a renin-angiotensin system inhibitor or a diuretic were the main pharmacodynamic interactions observed in drug-induced acute kidney injury. The combined use of two antithrombotic agents, such as an oral anticoagulant and an antiplatelet agent, and the combination of an oral anticoagulant with a selective serotonin reuptake inhibitor were the drug interactions most often observed in bleeding adverse drug reactions. Therefore, understanding the association of eGFR with adverse drug reactions helps in systematically assessing the risk/benefit ratio of anticoagulant and antiplatelet drugs for CKD.

The alterations of the pharmacodynamics and pharmacokinetics of anticoagulants in kidney failure make the patients’ management more challenging [54]. Previous studies reported that the changes in intestinal function in CKD influence the bioavailability of drugs. These alterations may be due to the reduction in first pass metabolism as a result of inhibition of some isoforms of cytochrome P450 in the enterocyte, or the decreased expression of intestinal membrane transporters (P-glycoprotein and multidrug-resistance-related protein), which contributes to enhancing the bioavailability of drugs in CKD [136,137]. The CYP3A4 isoform of cytochrome P450 is an enzyme that converts ticagrelor to its major active metabolite (AR-C124910XX). Molecules having a weight < 1000 g/mol have higher intestinal absorption. The molecular weight of ticagrelor is 522.567 g/mol [106]. It was reported in a recent study that ticagrelor exhibited higher inhibition of platelet aggregation than clopidogrel in CKD patients with stage 4–5 [102] because, after absorption, two-thirds of ticagrelor remains active and is converted to an active metabolite, des-ticagrelor (AR-C124910XX), by CYP3A4\5 [138]. Ticagrelor and des-ticagrelor result in the reversible blockage of ADP binding to the P2Y_12_ receptor on platelets. On the other hand, since clopidogrel is a pro-drug, 85% of clopidogrel is metabolized to inactive metabolite after absorption. The residual 15% is converted to 2-oxo-clopidogrel, with subsequent conversion to the active metabolite (R-130964) by CYP2C19, irreversibly blocking the binding of ADP to the P2Y_12_ receptor [139]. Similarly, the study of Nishi et al. [60] concluded that the antiplatelet activity of clopidogrel is reduced in CKD patients compared to non-CKD, whereas prasugrel consistently decreased the platelet reactivity regardless of mild to moderate CKD. The variation of the antiplatelet response of clopidogrel across individuals is due to the highly polymorphic nature of the *CYP2C19* gene [140]. *CYP2C19* loss of function allele carrier patients are more affected by thrombotic risk due to lower levels of overall active clopidogrel compared to non-carriers; this trait in combination with higher on-treatment platelet reactivity in CKD could increase the risk of thrombotic events to a greater extent, despite treatment [141,142]. The use of alternative oral P2Y_12_ inhibitors such as ticagrelor or prasugrel, independent of CYP2C19-based activation, improves high on-treatment platelet reactivity and reduces ischemic events in patients with *CYP2C19* loss of function alleles. However, the universal use of these potent P2Y_12_ inhibitors causes bleeding complications. Implementation of the precision medicine strategy based on *CYP2C19* genetic testing may offer a more balanced therapeutic approach to reduce the ischemic and bleeding risks by prescribing ticagrelor or prasugrel to *CYP2C19* loss of function allele carriers and clopidogrel to non-carriers [140].

A post hoc analysis of the TAILOR-PCI study led by Mathew et al. [141] reported that *CYP2C19* genotype-guided escalation of P2Y_12_ inhibitor therapy showed no reduction in primary outcomes, including CV death, stroke, myocardial infarction, stent thrombosis, and severe recurrent coronary ischemia in CKD, whereas it was more effective in non-CKD patients. However, there was no association between genotype-guided strategies, with increased bleeding risk in CKD. The reason for the limited benefits of genotype-guided strategies in CKD is not clearly understood. However, it could be due to high on-treatment platelet reactivity and other factors such as uremic toxins in CKD compared to non-CKD patients. Further, the author suggested that limited representation of the CKD patients in cardiovascular trials may make them resistant to implementation strategies, which suggests the need for more studies to understand the mechanism behind this resistance in this population. On the other hand, Thomas et al. [142] evaluated the association between ABCD-GENE (age, body mass index, chronic kidney disease, diabetes, and *CYP2C19* genetic variants) score and the effectiveness of clopidogrel vs. alternative P2Y_12_ inhibitor (prasugrel or ticagrelor) therapy after PCI. An ABCD-GENE score ≥ 10 predicts reduced clopidogrel effectiveness. Their findings indicated no significant difference in major atherothrombotic events with alternative therapy vs. clopidogrel among patients in both groups with scores < 10 and ≥10. However, alternative therapy resulted a lower risk of major atherothrombotic events among *CYP2C19* loss of function allele carriers in both groups, whereas clopidogrel was as effective as an alternative therapy in non-carriers with scores < 10. This study suggests the preference of clopidogrel over alternative therapy in non-carrier patients with scores < 10 and consideration of alternative therapies over clopidogrel for *CYP2C19* loss of function allele carrier patients regardless of ABCD-GENE score.

Abnormal PGI_2_ and thromboxane production due to endothelial dysfunction in CKD may decrease the aspirin ability to inhibit COX-1 and would cause the higher prevalence of aspirin resistance in these patients [100]. The study of Binder et al. [61] suggested that reduction in integrin α_IIb_β_3_ activity and platelet aggregation in CKD patients on HD is not only due to antiplatelet therapy. In their study, low daily doses of aspirin (75 mg) showed no additional effect on platelet activation and aggregation; the reason behind this could be aspirin resistance, which is common in HD patients. The association between carbamylation by uremic toxin and aspirin resistance is unknown; however, carbamylation may affect aspirin treatment by reducing its bioavailability or elevating platelet turnover.

Mörtberg et al. [66] reported that despite using DAPT such as P2Y_12_ receptor antagonists (clopidogrel, prasugrel, or ticagrelor) and aspirin, there was still elevated levels of MPs with the progression of CKD; this may be due to the lower efficacy of P2Y_12_ receptor antagonists in CKD patients. The antiplatelet therapy in CKD is still challenging due to the impact of renal failure on the efficacy of antiplatelet agents, which predispose the patients to a higher risk of adverse events [143]. Due to the insufficient effects of antiplatelet drugs, CKD patients develop high on-treatment platelet reactivity, which causes a high incidence of CV events and mortality [144]. In diabetes mellitus and CKD patients treated with standard DAPT, high post-treatment platelet reactivity was observed in ESRD but not in moderate CKD. This may be because of the existence of a threshold after which renal failure affects platelet reactivity [66].

The bioavailability of the DOACs is complicated by inter-patient variability, including different CKD stage, gastrointestinal physiology, comorbid conditions, pharmacogenetic factors, drug interaction, and body weight, which subsequently limit the optimal dosing of DOACs for CKD patients [107].

All studies of DOACs excluded ESRD patients because of the influence of kidney function on the pharmacokinetics of drugs, predisposing patients to a lower renal clearance of drugs and consequent major complications [108].

Warfarin binds with albumin, and the hepatic clearance of warfarin is reduced with decreased renal function. Hypoalbuminemia and deteriorating renal function increase the bioavailability of warfarin, resulting in a supratherapeutic international normalized ratio [110]. Interindividual variability in warfarin dosing is due to polymorphisms in vitamin K epoxide reductase gene and cytochrome P450 type 2C9 (CYP2C9), which account for 25% and 10%, respectively. The involvement of the vitamin K reductase genotype in the conversion of vitamin K epoxide to vitamin K could make it the best predictor of warfarin dose. *CYP2C9* alleles including CYP2C9*2 and *3 carriers are poor metabolizers compared with the wild-type CYP2C9*1 allele carriers [113]. DOACs demonstrate a better safety profile in comparison to warfarin, but the benefit/risk ratio of these anticoagulants still needs to be confirmed in the setting of severe renal failure. Moreover, the strategies to mitigate other pharmaceutical-related risk should be implemented to determine safety and outcomes in CKD patients [135].

## 6. Conclusions

Patients with CKD are highly susceptible to thrombotic and bleeding risk, and they are exposed to a higher risk of CV events, morbidity, and mortality. The characteristics of patients and treatment modalities cause variation in clinical presentations of hemostatic dysfunction in ESRD patients. Therefore, understanding the association among renal function, the pathophysiology of cardiorenal syndrome, endothelial dysfunction, platelet activity, endothelial and platelet MPs, and the evaluation of potential circulating biomarkers is essential to develop effective therapeutic strategies for CKD patients. Further studies are needed to investigate the alterations of hemostasis in dialysis patients, which subsequently help clinicians in choosing favorable dialysis modalities and antithrombotic treatment for this group of patients, to balance the bleeding and thrombotic risk.

## Figures and Tables

**Table 1:** Examples of modified dialyzer membranes with high hemocompatibility and performance efficiency.

Membrane	Uremic Toxin Removal (%)	BSA Retention (%)	Hemolysis Ratio (%)	PRT (s)	aPTT (s)	References
CA/PEG/PVA	93	96	3.2	300	-	Azhar et al. [18]
PES/TA/α-LA	96	99.7	0.3	297	80	Wei et al. [83]
PES/G/E-TA	54	93	0	-	45	Xu et al. [130]
NZ/PCNF	59	-	<2	-	-	Yang et al. [16]
PES/AR	-	>90	0.1	-	-	Hou et al. [134]
PES/PVP/PAA	-	-	0	>10	>60	Zhi et al. [132]

## Data Availability

No new data were created.
